# Microsphere-based interferometric optical probe

**DOI:** 10.1038/s41467-018-07029-9

**Published:** 2018-11-01

**Authors:** Yongjae Jo, Junhwan Kwon, Moonseok Kim, Wonshik Choi, Myunghwan Choi

**Affiliations:** 1 Department of Biomedical Engineering, Sungkyunkwan University, Suwon, 16419 Republic of Korea; 2 Center for Neuroscience Imaging Research, Institute for Basic Science, Suwon, 16419 Republic of Korea; 3 Center for Molecular Spectroscopy and Dynamics, Institute for Basic Science, Seoul, 02841 Republic of Korea; 4 Department of Physics, Korea University, Seoul, 02841 Republic of Korea

## Abstract

Fluorescent optical probes have rapidly transformed our understanding of complex biological systems by providing specific information on biological targets in the natural living state. However, their utility is often limited by insufficient brightness, photostability, and multiplexing capacity. Here, we report a conceptually new optical probe, termed ‘reflectophore’, which is based on the spectral interference from a dielectric microsphere. Reflectophores are orders-of-magnitudes brighter than conventional fluorophores and are free from photobleaching, enabling practically unlimited readout at high fidelity. They also offer high-degree multiplexing, encoded in their optical size, which can be readily decoded through interferometric detection with nanoscale accuracy, even in turbid biological media. Furthermore, we showcase their biological applications in cellular barcoding and microenvironmental sensing of a target protein and local electric field.

## Abstract (web summary)

Tagging and tracking cells with multiplexed labels can help study complex cellular behaviors in living systems. Here, Jo et al. propose and demonstrate the use of Fabry-Perot-like resonances in dielectric microspheres as such a label and call these reflectophores.

## Introduction

The introduction of optical probes has revolutionized our understanding of biological systems by providing unprecedented structural and functional information on specific biological targets in living milieu^1–3^. Among various contrast mechanisms, fluorescence is undoubtedly a current gold standard^4^. A vast library of fluorescent probes is readily available to visualize specific biological targets (e.g., ions, proteins, and cells) in subcellular-level precision and is rapidly expanding^5–7^. As with all techniques, however, fluorescence has weaknesses. Most importantly, its broad spectral emission limits the number of distinguishable entities (i.e., multiplexing) to typically 3–4 (ref. ^8^). Moreover, photobleaching impedes continuous observation over extended periods^9^. These factors pose limitations to experimental design and utility.

To complement these weaknesses, there have been continued efforts to develop new optical probes. In particular, many strategies have been proposed to enable high-degree multiplexing, such as spectral unmixing^10^, fluorescence lifetime^11^, barcoded particles^12–16^, and combinatoric labeling^17,18^. However, these techniques have not been successfully adopted widely because they necessitate either complex fabrication processes or decoding optic systems. Moreover, limited contrast and sensitivity in scattering biological tissues often hampers wide biomedical utilization. Recently, microlasers have received great attention due to their ultrahigh multiplexing capability^19–21^. Notably, whispering gallery lasers provide efficient lasing (high *Q*-factor) in micro-scale resonators, such as spheres, rings, and toroids. Their small form factor even enables the loading of various biocompatible microlasers into living cells and tissues^22,23^. However, their size is comparable to that of cells, which likely interferes with cellular signaling pathways. Moreover, ambient fluctuation of the intracellular refractive index destabilizes the lasing mode, and photobleaching of the gain medium limits the period of observation.

Here we introduce a brand-new optical probe, termed “reflectophore”, based on spectral interference from subcellular-scale dielectric microspheres. Compared to conventional fluorophores, reflectophores provide superior brightness, negligibly lower photobleaching, and orders-of-magnitude higher multiplexing. In contrast to previous interferometric approaches^24,25^, reflectophores have a rotation-invariant spherical design providing reliable three-dimensional detection even in biological media and possess variety of functionalization capacities via material engineering. We report comprehensively on reflectophores from their theoretical background to their biological utilities in cellular barcoding and microenvironmental sensing.

## Results

### Concept of reflectophore

**Fig. 1:**
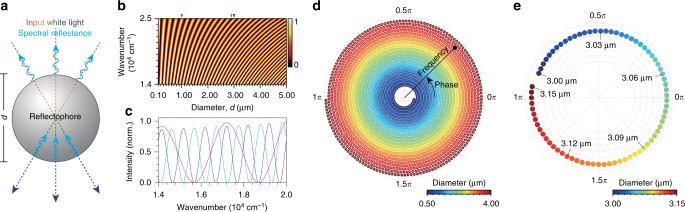
Concept of reflectophore. **a** Schematic representation of a reflectophore. A dielectric microsphere can serve as a reflectophore through Fresnel reflections and interference from a broadband input source. Each reflectophore is distinguishable by its reflectance spectrum, which encodes its diameter (*d*) with nanoscale precision. **b** Simulated reflectance spectra of polystyrene-based reflectophores. Colorbar, normalized reflectance. **c** Representative simulated reflectance spectra of the polystyrene-based reflectophores (magenta: 1.00 μm, cyan: 2.90 μm, and blue: 3.00 μm). Note that their diameter is encoded in the frequency and phase of the reflectance spectrum. **d** Phasor representation of the simulated spectra in **b**. Angle and radial distance correspond to phase and frequency, respectively. Phase is set to 0*π* at 500 nm. **e** A representative phasor representation for polystyrene-based reflectophores with diameters ranging from 3.00 to 3.15 μm at an interval of 2 nm

Optical rays focused at the center of a dielectric sphere are geometrically analogous to parallel rays that incide orthogonally onto a planar structure with a thickness that matches the sphere’s diameter (Fig. 1a). Based on this geometric analogy, we performed numerical simulations for thin-film interference on polystyrene-based microspheres at various diameters (*d* = 1–20 μm)^26^ (Supplementary Note 1, Supplementary Figs. 1 and 2). We verified by using a more detailed vector-diffraction model that the simplified thin-film model provides computational efficiency and reliable estimation of reflectance spectra (Supplementary Fig. 3). We observed that the simulated reflectance spectra are sinusoids when plotted against wavenumber, which is the inverse of wavelength (Fig. 1b, c, Supplementary Fig. 4 and Supplementary Note 2). Notably, the diameter is uniquely identifiable using the frequency and phase of the spectrum. An increase in diameter leads to a higher spectral frequency and also introduces a spectral shift toward red (i.e., bathochromic shift). This relationship can be clearly represented in a phasor diagram in which radial distance and angle correspond to frequency and phase, respectively (Fig. 1d). In phasor representation, an increase in diameter resulted in an outward spiral trajectory. The phase encoded information on the diameter more sensitively than frequency. For example, a change of 10 nm in a microsphere of ~3 μm in diameter exhibited a *π*/8 phase shift, which can be readily detected (Fig. 1e).

We reasoned that the reflectance spectra of microspheres may serve as optical labels, namely, reflectophores. Reflectophores enable high-degree multiplexing by allowing a precise readout of their diameter. For example, a precision of 3 nm would provide a multiplexing of over 300 within a diameter range of 1 μm, which is in stark contrast to fluorophores, which have a typical multiplexing of 3–4^8^.

### Proof-of-concept

**Fig. 2:**
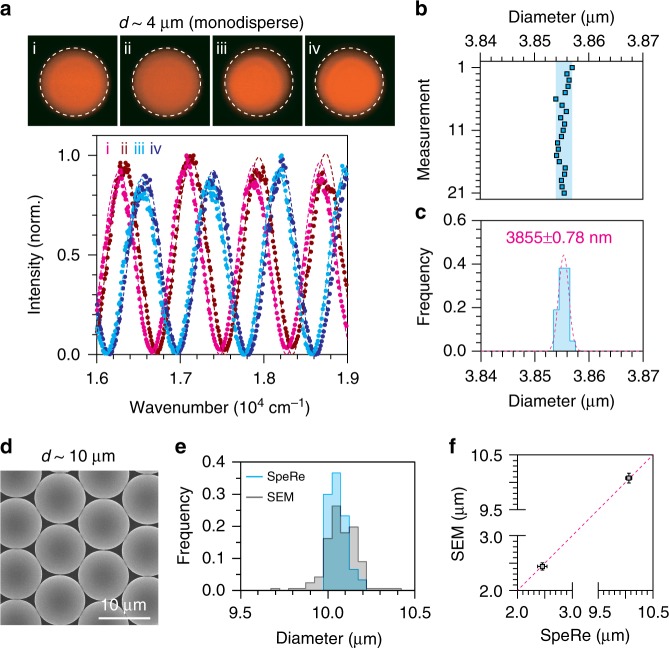
Proof-of-concept. **a** SpeRe measurements on polystyrene-based fluorescent reflectophores with a nominal size of 4 μm. The measured diameters are 3.756 μm for “i”, 3.742 μm for “ii”, 3.693 μm for “iii”, and 3.684 μm for “iv”. Diameters (*d*) were quantified by finding the best-fit simulated spectrum (*R*^2^: 0.99 for “i”, 0.98 for “ii”, 0.98 for “iii”, and 0.98 for “iv”). The upper panel shows fluorescent images on the corresponding reflectophores. **b**, **c** Measurement precision. A single silica-based reflectophore with a nominal size of 4 μm was repeatedly measured through SpeRe. Precision in standard deviation was ±0.78 nm (*n* = 21 measurements). The shaded blue area in **b** indicates maximum deviation. The value in **c** is presented as mean ± standard deviation. **d**–**f** Validation of the reflectophore measurements using a scanning electron microscope (SEM). The SpeRe measurements on multiple reflectophores were compared with the SEM measurements (unpaired Student’s *t*-test; monodisperse beads: 10.061 ± 0.0531 μm and 10.078 ± 0.0905 μm for SEM (*n* = 167 beads) and SpeRe (*n* = 30 beads), respectively, *p* = 0.147; polydisperse beads: 2.440 ± 0.065 μm and 2.457 ± 0.087 μm for SEM (*n* = 151 beads) and SpeRe (*n* = 30 beads), respectively, *p* = 0.206; The values are presented as mean ± standard deviation). The error bars in **f** represent standard deviation. Source data are provided as a Source Data File

To test the feasibility of reflectophores, we set up a spectral reflectometry (SpeRe) system by coupling a supercontinuum laser to a confocal microscope and directing the reflected light to an array spectrometer^27,28^ (Supplementary Fig. 5). To reliably acquire a reflectance spectrum at the geometric center of the microsphere, we volumetrically scanned around the center, and the spectrum with the maximum reflectance was selected (Supplementary Fig. 6). Following this procedure, we first tested the measurement precision of our SpeRe system (Fig. 2a). For monodisperse microspheres (~4 μm), our SpeRe system unambiguously differentiated between four randomly chosen microspheres, which had a maximum variability of only ~70 nm. By contrast, conventional fluorescence failed to distinguish between subtle differences in size, as expected due to the physical diffraction limit (~200 nm). The precision obtained by 21 repeated measurements on a single silica microsphere was ±0.78 nm (Fig. 2b, c). The maximum difference in the measured diameters was 3 nm, corresponding to the theoretical limit arising from our system’s spectral resolution of 0.6 nm. This precision can provide high-degree multiplexing of >300 within a size range of 1 μm.

To further validate the accuracy of SpeRe measurements, we used a scanning electron microscope (SEM). We prepared two types of microspheres (*d* ≈ 2.6 μm and 10 μm), each fabricated from the same batch, and measured their diameter distribution (i.e., sample mean) with either SpeRe or SEM. In these measurements, we obtained nearly identical diameter distributions (Fig. 2d–f). We further confirmed that the correlative measurements with the SpeRe and SEM on the same reflectophores provided nearly identical sizes, verifying the nanoscale accuracy of the SpeRe measurements (Supplementary Fig. 7).

### Optical characterization

In fluorescence, brightness is determined by multiplication of the absorption cross section (*σ*) and the quantum yield (*Φ*). Typically, quantum yield is high (e.g., *Φ* = 0.92 for fluorescein) but absorption cross-section is tiny (e.g., *σ* = 0.0121 nm^2^ for fluorescein), requiring a high concentration of fluorophores (>1 μM) for attaining practically detectable emissions^29,30^. In reflectophore, brightness is determined by the refractive indices of the internal material and the external media, described in Fresnel equation. In the case of polystyrene-based microspheres (*n* ≈ 1.59), their total reflectance in water (*n* ≈ 1.33) is ~3.5%, which is ~700 times brighter than the fluorescence emitted from 100 μM fluorescein (Supplementary Note 3).

**Fig. 3:**
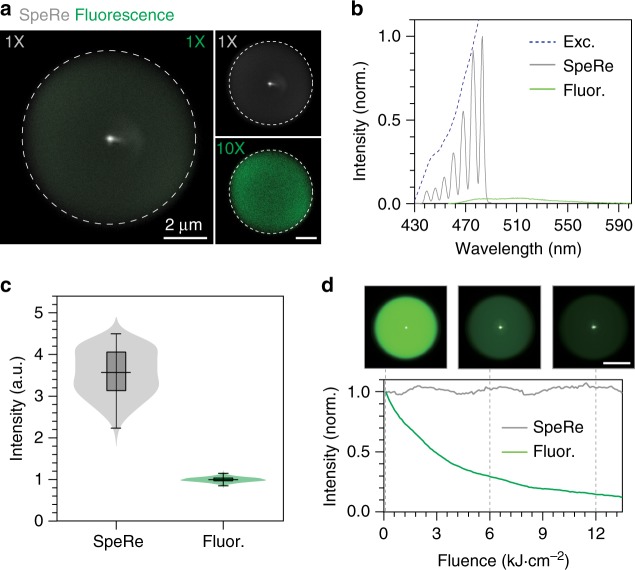
Comparison of a reflectophore with a fluorophore. **a** Simultaneous spectral reflectance (SpeRe) and fluorescence imaging on a fluorescent reflectophore. The left panel shows the merged image of SpeRe (gray) and fluorescence (green), measured at the same detector gain (1× for SpeRe and fluorescence). Note that fluorescence is nearly invisible due to its orders-of-magnitude lower brightness. The right panel separately shows SpeRe and fluorescence images at the indicated detector gain (1× for SpeRe, 10X for fluorescence). Scale bar, 2 μm. **b** Measured spectra for reflectance (gray) and fluorescence (green) with input beam focused at the centroid. *Exc* excitation light, *Fluor* fluorescence. **c** Quantification of the total optical intensity in **b** presented as a violin plot and a box-and-whisker plot (center line, median; box limits, upper and lower quartiles; whiskers, min and max; *n* = 121 pixels for SpeRe; *n* = 2700 pixels for fluorescence). Source data are provided as a Source Data File. **d** Photobleaching kinetics. A fluorescent reflectophore was serially imaged by both SpeRe (gray) and fluorescence (green). Note that fluorescence exponentially decays over time whereas reflectance stays constant. Scale bar, 4 μm

To verify this, we imaged a fluorescent microsphere with a nominal diameter of 8 μm by both reflectance and fluorescence techniques. It is worth mentioning that this microsphere was highly fluorescent, equivalent to ~5 mM fluorescein. The input laser was tuned close to the excitation maxima (465 ± 20 nm), and the emitted light was epi-collected either by photomultiplier tubes (for imaging) or a spectrometer (for spectral analysis). When imaged at the same detector gain, fluorescence was nearly invisible compared to reflectance (Fig. 3a). Reflectance was over ~10 times brighter in total emission than fluorescence (Fig. 3b, c). Theoretically, 8-μm reflectophores are 14 times brighter than 5 mM of fluorescein, which agrees well with our experimental results. From a practical standpoint, reflectophores are ~14,000 times brighter than a typical fluorescent cell labeled with 10 μM eGFP (*Φ* = 0.6, *σ* = 0.0091 nm^2^)^31^. It should be noted that reflectance is localized only to the centroid, whereas fluorescence is evenly distributed, and the above comparison was made when the detection was at the centroid. A fluorescent signal linearly scales with the number of integrated fluorescent pixels and thus may be comparable or greater than the reflectance signal if off-centered signals are considered.

An additional compelling advantage of reflectophores over fluorophores is that reflectophores do not involve excited electronic states and, thus, are free from photobleaching. In reflectophore, optical signal is generated by its structure (i.e., structural color), therefore the signal is highly stable, unless the material is photothermally deformed. For example, reflectophores made of polystyrene would not deform at the laser power used in our protocol (~20 μW) because the estimated temperature change is safely below the glass-transition temperature (Supplementary Note 4). On the contrary, fluorophores are vulnerable to photobleaching at relatively low fluence^32^. To demonstrate this, we performed repeated serial imaging of reflectance and fluorescence on a fluorescent microsphere. Indeed, we observed that the reflectance signal was nearly constant for over an hour under excitation at 60 μW. By contrast, fluorescence decreased exponentially with a fluence half-life of 3.5 kJ cm^−2^.

**Fig. 4:**
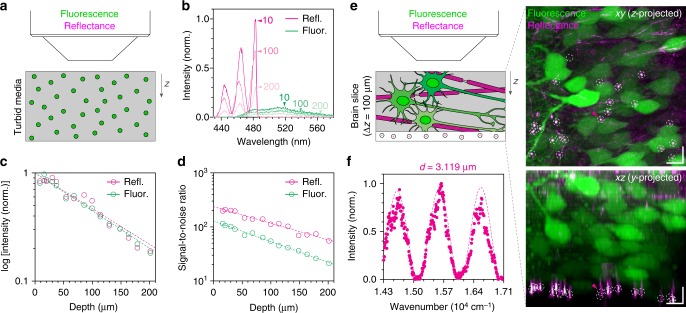
Reflectophore in turbid media. **a** Setup of phantom study. The phantom was prepared by mixing fluorescent reflectophores (*d* ≈ 3 μm and 8 μm) in a scattering medium (alumina 0.2% (w/w) in PDMS). **b** Representative reflectance (magenta) and fluorescence (green) spectra for reflectophores in the scattering media. The numbers with arrowheads indicate depths in μm. **c** Quantification of attenuation length. Dashed lines indicate the best-fit to the exponential decay (*R*^2^: 0.94 for “Refl.” and 0.96 for “Fluor.”). **d** Quantification of signal-to-noise ratio. Dashed lines indicate the best-fit to the exponential decay (*R*^2^: 0.97 for “Refl.” and 0.99 for “Fluor.”). **e** Reflectophore in brain tissue. A brain slice prepared from a Thy1-YFP mouse is overlaid on top of polystyrene-based reflectophores (*d* ≈ 3 μm) and imaged by fluorescence (green) and reflectance (magenta) in a *z*-stack. The fibrous structures in the reflectance image are myelinated axons because of their unique multilayered cytoarchitecture. The dotted circle indicates the reflectophore at a depth of 100 μm from the tissue surface. To compensate the depth-dependent signal attenuation, intensity was normalized for each slice. Scale bar, 10 μm in *xz*. **f** Reflectance spectrum measured from the reflectophore in **e**. Dotted line is the best-fit simulated data (*d* = 3.119 μm) to the measured spectrum (*R*^2^ = 0.96)

We further questioned whether reflectophores are practically applicable in scattering biological media. We compared reflectance with fluorescence in a scattering polymer matrix encapsulating fluorescent reflectophores (Fig. 4a). Using acquired spectra across depth, we obtained a 1/*e* attenuation length and signal-to-noise ratio (SNR) (Fig. 4b). The SNR was obtained from the average intensity divided by the standard deviation of background noise. In agreement with our previous characterization (Fig. 3), reflectance exhibited higher signals than fluorescence, and their attenuation lengths determined by depth-dependent signal decay were similar (Fig. 4b,c). Notably, reflectophores showed higher SNRs, conceivably because of the concentrated signal at the centroid (Fig. 4d). To further evaluate the applicability in biological tissues, we mounted reflectophores beneath a cortical brain slice of a Thy1-YFP mouse and performed a SpeRe measurement (Fig. 4e). Consistent with the tissue phantom study, we obtained reliable spectral measurements for reflectophores under a 100-μm-thick tissue slab (Fig. 4f). The results collectively indicated that reflectophores can be adapted in turbid biological media.

### Intracellular loading and tracing

**Fig. 5:**
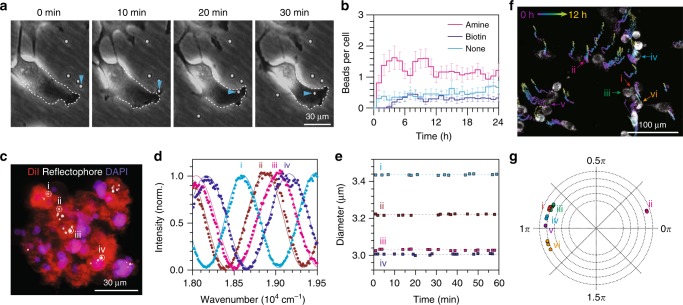
Intracellular reflectophores. **a** Time-lapse phase-contrast images of live Hela cells in culture engulfing reflectophores. Arrowheads indicate the reflectophore under endocytosis. The dotted line demarcates the cell’s margin. **b** Population kinetics of intracellular uptake for surface-functionalized polystyrene reflectophores (*d* ≈ 3 μm). The number of samples for each group: *n* = 45 cells for “amine”, *n* = 66 cells for “biotin”, and *n* = 50 cells for “none”. The error bar represents standard error of the mean. **c** A long-term 3D spheroid culture. Hela cells loaded with reflectophores were subsequently cultured over several days to form a tumor spheroid. **d** Representative spectral measurements on intracellular reflectophores annotated in **c**. The measured diameters are 3436 nm for “i”, 3212 nm for “ii”, 3029 nm for ‘iii’, and 3006 nm for “iv”. The solid sinusoidal curves are the best-fit simulation spectra (*R*^2^: 0.99 for “i”, 0.98 for “ii”, 0.99 for “iii”, and 0.97 for “iv”). **e** Measurement precision for intracellular reflectophores. Individual intracellular reflectophores annotated in **c** were traced over 60 min. The measured diameters are 3436 ± 2.6 nm for ‘i’, 3212 ± 3.1 nm for ‘ii’, 3029 ± 2.9 nm for ‘iii’, and 3006 ± 2.6 nm for “iv” in mean ± standard deviation. The dotted lines indicate the means. **f** Barcoded cell tracking over 12 h with intracellular reflectophores. Grayscale image indicates MitoTracker fluorescence and pseudo-colored traces represent time-series tracking of individual reflectophores over time. **g** Phasor representation of six representative reflectophores measured at multiple time points. The measured diameters are 3169 ± 0.2 nm for “i”, 3379 ± 1.8 nm for “ii”, 3015 ± 0.2 nm for “iii”, 3165 ± 0.7 nm for “iv”, 3155 ± 2.1 for “v”, and 3147 ± 0.9 nm for “vi” in mean ± standard deviation

Due to their micron-scale size, conventional intracellular delivery methods, such as passive diffusion or electrophoresis, cannot be used with reflectophores. Alternatively, it has been reported that both phagocytic and non-phagocytic cells can actively uptake microparticles via endocytosis^33–35^. This active phagocytic process has been adopted for introducing intracellular microcavities (*d* = 5–20 μm) into live cells^20,22,23^. To test its applicability on smaller-scale microspheres, we treated polystyrene reflectophores (*d* ≈ 3 μm) with different surface coatings (bare, amine, and biotin) to multiple cell lines in culture and took time-series phase-contrast images over 24 h (Fig. 5a). We validated that serial phase-contrast images provide a robust quantification of intracellular uptake by showing that the cell-impermeant fluorescent streptavidin does not stain intracellular biotinylated reflectophores (Supplementary Fig. 8). To our surprise, we observed that the surface coating significantly affected uptake efficiency: amine-functionalized reflectophores showed an over two-fold higher uptake compared to bare and biotinylated reflectophores (Fig. 5b). This result is conceivably due to the positive surface charge of the amine-coated reflectophore (pK_a_ ~10)^36^, which produces attractive electrostatic interaction with the negatively charged cell membrane^37^.

In the time-domain analysis, the number of intracellular reflectophores showed one-phase exponential association kinetics with a half-life of ~30 min (Fig. 5b). As expected, cell division introduced the segregation of intracellular reflectophores into two daughter cells. The measured doubling time in the presence of intracellular reflectophores was ~21 h, which is similar to control cells without reflectophores^38,39^. Although rare, we also observed that reflectophores escaped the cell (i.e., exocytosis), sometimes during cytokinesis (Supplementary Fig. 9). After ~3 h, the uptake was balanced by exocytosis and cell division, reaching a steady-state value of ~1.2 amine-coated reflectophores per cell. We did not observe any signs of cellular damage such as membrane permeabilization, blebbing, or apoptosis by intracellular reflectophore in three cell lines (Supplementary Fig. 10). Collectively, these results illustrate that reflectophores can be rationally designed to be internalized into cells, and intracellular reflectophores do not significantly interfere with cellular physiology.

Next, we questioned whether differently sized reflectophores could serve as multiplexed labeling for tracing individual cells, as also proposed in intracellular microlasers^20^. To address this, we prepared multicellular spheroids using reflectophore-loaded cells (Fig. 5c). We then tested whether the spectral measurements were reliable on intracellular reflectophores by repeated measurements on four individual reflectophores over a period of 1 h (Fig. 5d, e). Precision was measured to be ±3 nm, which is somewhat compromised when compared to the cell-free condition (±1.5 nm), presumably due to the heterogeneity of intracellular microenvironments. Still, this precision would offer multiplexing of >100 within a size range of 1 μm.

Last, we tested the potential of optical barcoding for tracking migrating cells (Fig. 5f). We pre-loaded polystyrene reflectophores (*d* ≈ 3 μm) to B16F10 melanoma cells by co-incubation for 24 h and replated on a Matrigel-coated dish. As reported previously, the cells plated on Matrigel formed capillary-like intercellular networks within 24 h^40^. We observed no significant change in migration activity by intracellular reflectophores (Supplementary Fig. 11). We next tracked individual migrating cells in the process of forming capillary-like structures with reflectophore barcoding. The spectral features for multiplexed barcoding were stably maintained in each migrating cell over 24 h as apparent in the phasor diagram (Fig. 5g).

### Microenvironmental sensing

**Fig. 6:**
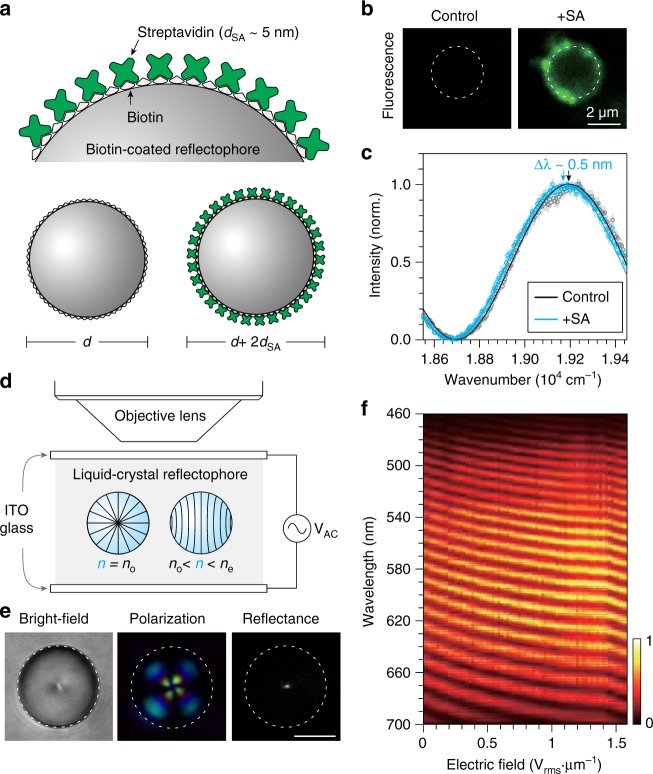
Microenvironmental sensing. **a** Schematic illustration for the binding of streptavidin on a biotin-functionalized reflectophore. Upon binding, diameter increases by ~10 nm. **b** Fluorescence images before and after loading fluorescent streptavidin. Note that fluorescence was localized specifically at the surface of the reflectophore. **c** SpeRe measurements on control and streptavidin-bound reflectophores. The arrows indicate the spectral peaks. Data are represented as mean (open circle) and the standard deviation (shaded region) was acquired from 30 repeated SpeRe measurements. The solid lines are the best-fit simulated spectra (*R*^2^ in mean ± standard deviation: 0.98 ± 0.005 for “control”, 0.98 ± 0.005 for “+SA”). **d** Experimental setup for sensing electric field with a liquid-crystal reflectophore. Nematic liquid-crystal droplet is immersed in PDMS and sandwiched between conductive ITO glasses coupled to a function generator. **e** Liquid-crystal reflectophore imaged by bright-field, polarization, and reflectance microscopy. Scale bar, 4 μm. **f** Spectral reflectance of liquid-crystal reflectophore in response to applied electric field. Color bar represents normalized reflectance

We reasoned that the nanoscale precision realized may enable the sensing of macromolecules adhered to the reflectophores via specific biological interactions. Molecular adsorption forms an additional dielectric thin-film layer with a thickness corresponding to the size of the macromolecule, which can be detected by SpeRe (Fig. 6a). For example, streptavidin, a protein showing specific biological binding to biotins (*k*_d_ = 400 pM), has a refractive index of 1.50 and a size of ~5 nm^41–44^. If a biotinylated reflectophore is coated by streptavidin, its diameter increases by 10 nm, and the refractive index of the streptavidin layer depends on the surface density of the protein. In our numerical simulation, we estimated that the binding of streptavidin leads to a spectral redshift (i.e., bathochromic shift) of up to ~1 nm, depending on the protein density (Supplementary Fig. 12). To test its feasibility, we performed SpeRe measurements on a biotinylated reflectophore (*d* ≈ 3 μm) before and after introducing streptavidin. We used fluorescent streptavidin to independently confirm its binding to the reflectophore (Fig. 6b). After binding, we observed a robust redshift of 0.57 ± 0.1 nm (*n* = 11 microspheres), corresponding to an effective surface protein density of ~52% (Fig. 6c and Supplementary Fig 13). This protein density is consistent with the independent estimation based on fluorescence intensity (Supplementary Note 5).

The information encoded by reflectophores is the optical distance, thus, the change in refractive index, as well as size, can be detected at high precision. To demonstrate this alternative sensing principle, we prepared a liquid-crystal reflectophore via self-assembly of a nematic liquid crystal into a droplet in a polymer matrix (Fig. 6d). The sample was then sandwiched between conductive indium-tin-oxide glasses and connected to a function generator to control the external electric field during optical sensing. When there was no external electric field, liquid-crystal reflectophores exhibited radially ordered structures, which was apparent in bright-field and polarization microscopy (Fig. 6e). In this molecular geometry, the focused incoming beam senses an ordinary refractive index (*n*_o_ ≈ 1.55) along the transversal axis of the liquid-crystal molecules^45^. With an increasing electric field applied, the internal liquid-crystal molecules are elastically reoriented and accordingly the effective refractive index increases because of the positive birefringence (*n*_e_ ≈ 1.74). In SpeRe measurements, we observed that the measured spectra shifted toward longer wavelengths at higher electric fields corresponding to the higher refractive index, consistent with the theoretical prediction (Fig. 6f).

## Discussion

We reported on the reflectophore, a novel interferometric optical probe that encodes information by optical distance (i.e., physical size and refractive index). Using off-the-shelf reflectophores and the SpeRe imaging system, we showed that the measurement precision for physical size in our protocol was ±1.5 nm in an aqueous medium and ±3.0 nm in intracellular environments. This precision can provide highly multiplexed cellular barcoding (>100 cells) as well as quantitative affinity-based molecular sensing using a surface-functionalized reflectophore. Furthermore, the variable refractive index of a liquid-crystal reflectophore enabled the sensing of local electric fields.

Our theoretical and experimental studies demonstrated that reflectophores have several compelling advantages. First, reflectophores have orders-of-magnitude higher brightness compared to typical fluorophores because of the high reflective cross-sections of their spherical geometry. The bright signal localized at the centroid provides high SNR for reliable spectral detection even in scattering media (Fig. 4). The spherical shape also ensures orientation-independent readouts for applications in three-dimensional tissues, which is in stark contrast to previously reported barcodes requiring a certain readout angle^13^. Second, reflectophores exhibit negligible photobleaching. This enables a practically unlimited number of readouts as well as long-term information storage. Third, reflectophores are based on self-interference in a Fabry-Pérot etalon; therefore, they are resistant to dynamic changes in the external refractive index. Changes in medium composition, which are often unavoidable in applications in living systems, only affect the magnitude of reflectivity but not its spectral shape, thereby providing robust readouts (Supplementary Fig. 12). Last, as shown in our studies using a fluorescent microsphere, reflectophores can be synergistically integrated with existing fluorophores to take advantage of both modalities. The signals are easily separable in the spectral domain because of the Stokes shift in fluorophores and also in the spatial domain because of the center localization in reflectophores. This feature provides an additional degree-of-freedom in design, for example, to increase the multiplexing capacity for barcoding or to incorporate multiple sensors.

Reflectophores do, however, have shortcomings. Primarily, the size of reflectophores is typically 1–4 μm in diameter, which is orders-of-magnitudes larger than conventional fluorophores. Thus, reflectophores are not suitable for labeling individual proteins or subcellular organelles. In addition, the large size may interfere with cellular physiology when it is introduced into cells. Although we observed that intracellular reflectophores do not significantly interfere with viability, migration, and proliferation in multiple cell lines, interference to the biological process of interest should be verified for each experimental context. Applications in extracellular space in tissues or cell-free systems are relatively free from this issue. Next, for high-degree multiplexing, fabrication of various microspheres at nanoscale precision are required and is technically challenging. Ranges of nanotechnologies are available but may have low throughput and are costly^46,47^. As a potential solution, bulk polydisperse microspheres may be sorted based on our SpeRe-based readout, like the widely adapted fluorescent-assisted cell sorter. Last, our current readout system is rather slow as it involves volumetric scanning for locating the geometric center of a reflectophore. Since off-centered acquisitions are ignored, an advanced scanning algorithm to efficiently find the centers will dramatically accelerate acquisition speed. Alternatively, reflectophores could be physically aligned through optical or magnetic tweezers. In particular, optical tweezers would be easily integrable because the readout beam itself could exert optical forces to align the center of a microsphere toward the beam’s focus.

Although our studies only exemplified reflectophores made with certain materials (e.g., polystyrene, silica, and liquid crystal), any dielectric materials such as polymers and droplets can be broadly adopted. Recently, a plethora of hydrogels equipped with various functionalities were developed for bio-integration^48^. Notably, stimuli-responsive hydrogels can change their physical size in response to specific chemical or physical milieu such as pH, temperature, and electric field. Reflectophores made with these stimuli-responsive materials could function as bio-integrable sensors. It may also be possible to fabricate multilayered reflectophores to incorporate multi-functional sensing capabilities. Moreover, chemical engineering on the surface offers an additional route for functionalization such as affinity-based molecular sensing, improved biocompatibility, and targeting of a specific cell type^37^. We envision that the adoption of advanced knowledge in material science and chemistry will vastly expand the utilities of reflectophores.

## Methods

### Optical simulation

Numerical simulation on the reflectance spectra for various reflectophores was performed using a thin-film multilayer toolbox in MATLAB (https://sites.google.com/site/ulfgri/numerical/tftb)^26^. The spectral range was set to 400–700 nm at an interval of 0.3–0.03 nm. The refractive index spectra for water and silica was provided by the toolbox and that of polystyrene was adopted from a previous study^49^. To obtain the simulation database, the simulation was comprehensively performed on silica- and polystyrene-based reflectophores with diameters ranging from 1 to 20 μm at an interval of 0.1–1 nm.

### Optical setup

SpeRe measurements were performed on a customized galvanometer-based laser scanning microscope (6215H, Cambridge Technology; Supplementary Fig. 5). For the input source, a supercontinuum white light laser (EXB-6, NKT photonics) was attenuated using a neutral density filter (3.5 OD, Thorlabs), bandpass-filtered to 450–700 nm, and coupled to the microscope body (Ultima IntraVital, Bruker). Laser intensity measured at the objective back aperture was ~16 μW. Through a water-immersion objective lens (40×, 1.15 NA, Nikon), the laser beam was focused and volumetrically scanned around the center of the microspheres. The typical scanning dimensions were 9 × 9 × 10 pixels in XYZ with a lateral spacing of 100 nm and an axial spacing of 200 nm. Pixel dwell time was 30 ms. For fast scanning, scanning volume and pixel dwell time were reduced to 5 × 5 × 8 pixels and 5 ms, respectively. The reflected light was spatially filtered by a confocal pinhole (0.5 Airy unit) and collected by a spectrometer (Shamrock 303i, Andor) with an electron-amplifying charge-coupled detector (Newton, Andor). The slit was adjusted to 10 μm, providing a spectral resolution of ~0.6 nm with a grating of 150 lines mm^−1^. Prairie software (Bruker) was used for controlling the scanner and triggering the spectrometer.

### Data analysis

For each reflectophore, the SpeRe data were obtained by using volumetric scanning with 9 × 9 × 10 pixels in XYZ (∆_*xy*_ = 100 nm, ∆_*z*_ = 200 nm). The spectrum corresponding to the geometric center of the microsphere was acquired at the pixel with the maximum intensity (Supplementary Fig. 6). If the geometric center was not within the scanning volume, the dataset was excluded from the analysis. The spectrum was subsequently normalized by the reference spectrum obtained with a protected silver mirror (PF05–03-P01, Thorlabs) at the sample stage. The simulated spectrum best-fitted to the measured spectrum was chosen from the simulation database (*d* = 0.1–20 μm at 0.1-nm interval) based on Pearson’s correlation (calculated by a “corr” function in MATLAB).

### Optical characterization

Fluorescent microspheres (35-3, Thermo Fisher Scientific) immersed in 0.5% agarose gel (A4018, Sigma Aldrich) were used for optical characterization. To gauge fluorescence levels, we compared the fluorescence obtained with the microsphere with that of a known dye, fluorescein isothiocyanate-dextran (46944, Sigma), at the same optical gain and excitation intensity. For the measurement of brightness, we tuned the input source to its fluorescence excitation maxima (465 ± 20 nm, 30 μW) and acquired the spectrum of epi-collected signals after the confocal pinhole. To measure photobleaching, the microspheres were repeatedly imaged at an optical intensity of 60 μW for an hour (101.5 J cm^−2^ for each frame).

### Scanning electron microscope

For sample preparation, 3 μL of each microbead stock solution (72822-5ML-F, Sigma; PP-25-10, Spherotech) were dispensed on steel plates and dried in a fume hood. For correlative SEM-SpeRe imaging, the microspheres (*d* ≈ 10 μm) were affixed to an adhesive copper tape. After coating with platinum in a Gatan-682 precision etching and coating system, SEM images were taken on a field-emission SEM (JSM-7600F, JEOL) operating at 15 kV. A standard calibration kit (683–01C, Ted Pella) was used for scale calibration.

### Preparation of scattering samples

The scattering phantom was prepared by embedding 8 μm of fluorescent (35-3, Thermo Fisher Scientific) or 3 μm (80304, Sigma Aldrich) of bare reflectophores in PDMS (Sylgard 184, Dow Corning) mixed with 0.2% (w/w) aluminum oxide nanoparticles (~0.5 μm). The excitation source was adjusted to maximum fluorescence excitation (465 ± 20 nm, 60 μW). To compare the intensity and SNR attenuation of SpeRe and fluorescent, a depth-dependent image was acquired. The SNR was obtained by the mean intensity divided by the standard deviation of the background signal.

For tissue study, male or female Thy1-YFP transgenic mice aged 7–10 weeks old (The Jackson Laboratory, 003782) were cardiac perfused with phosphate-buffered saline (PBS) and 4% paraformaldehyde (PFA). The brain was extracted and post-fixed in 4% PFA for 24 h. The brain was sliced to 100–200 μm in thickness using a vibratome (VT1200S, Leica), and polystyrene reflectophores (*d* ≈ 3 μm) were sandwiched between the folded brain slices. All animal experiments were performed in compliance with institutional guidelines and approved by the subcommittee for research animal care at Sungkyunkwan University.

### In vitro studies

Three cell lines including HeLa, B16F10, and NIH3T3 were purchased from ATCC. Cells were maintained in Dulbecco’s modified Eagle’s medium supplemented with 10% fetal bovine serum for the HeLa and B16F10 cell lines and fetal calf serum for the NIH3T3 cell line and 1% antibiotics (anti-anti, Gibco) at 37 °C in 5% CO_2_. For the intracellular uptake assay, we used three types of polystyrene microspheres of ~3 μm in diameter: uncoated (80304-5ML-F, Sigma), amine-coated (AP-30-10, Spherotech), and biotin-coated. Microspheres were washed three times with PBS and were then treated to cells (8 × 10^5^ microspheres on 2.4 × 10^5^ cells in 35-mm culture dishes). Soon after, we placed the culture dish in a microincubator (37 °C, 5% CO_2_) and took phase-contrast images at 1-min intervals for 24 h using a wide-field microscope (DMi8, Leica). For the biotin-coated microspheres, we afterwards confirmed their intracellular localization by taking fluorescence images after adding 0.2 mg mL^−1^ of fluorescent streptavidin (S32354, Life Technologies), which is impermeable to cell membrane. For the spheroid culture, the cells encapsulating microspheres were trypsinized after 24 h of incubation, and 10-μL droplets of the cell suspension (2.4 × 10^5^ cells mL^−1^) were laid on the inner surface of a petri dish lid. After 3 days of incubation, the spheroids were stained with 20 μL mL^−1^ of DiI (D282, Thermo Fisher Scientific) and 2 μL mL^−1^ of DAPI (4',6-diamidino-2-phenylindole; D1306, Thermo Fisher Scientific) for 8 min and then imaged in our optic setup (Supplementary Fig. 5).

For the viability assay, 8 × 10^5^ beads were loaded on 2.4 × 10^5^ cells in a 35-mm culture dish. After incubation for 24 h, the cells were stained with 4 μM of ethidium homodimer-1 and 2 μM of calcein AM (L3224, Invitrogen) for 40 min and washed three times with a serum-free medium. For negative control, the cells were treated with 70% (v/v) methanol.

For the migration assay, 3.6 × 10^6^ amine-coated reflectophores were treated to 6 × 10^5^ B16F10 cells in a 35-mm culture dish. After 24 h of incubation, the reflectophore-loaded cells were stained with 500 nM of mitotracker (M7512, Invitrogen) for 40 min, washed three times with PBS, and plated on a Matrigel-coated dish (E1270, Sigma Aldrich). Cell tracking was started 4 h after the plating and continued for 24 h.

For the scratch-migration assay, 6 × 10^5^ B16F10 cells in a 35-mm culture dish were treated with 3.6 × 10^6^ biotin-coated reflectophores and incubated for 24 h. Negative staining of fluorescent streptavidin (0.2 mg mL^−1^) was used to confirm the intracellularly loaded reflectophores. Cellular injury was then performed using a pipette tip, typically resulting in a confined cellular detachment (~200 μm in width). Migration at the injured site was traced in a phase-contrast microscope (DMi8, Leica) with a microincubator at 1-min intervals for 24 h. The cell migration velocity was quantified using an MTrackJ plugin in ImageJ.

### Microenvironmental sensing

Biotin-coated microspheres with a diameter of ~3 μm were prepared by reacting biotinyl-N-hydroxysuccinimide (H1759, Sigma Aldrich) with amine-functionalized polystyrene microspheres (AP-30-10, Spherotech). After the reaction, which took place in PBS for an hour at room temperature (~25 °C), the biotin-coated microspheres were washed four times with PBS to remove unreacted reagents. The biotin-coated microspheres (8 × 10^4^ microspheres in PBS) were then placed on a customized sample chamber, composed of a micromesh (92315T101, McMaster-Carr) sandwiched by a porous membrane (pore size: 1 μm) and a glass coverslip. The microspheres were placed within the wells of the micromesh. We typically chose the microspheres adhered near the edge of the micromesh because of their positional stability even during solvent exchange. After the baseline spectral acquisition at a spectral resolution of 0.1 nm was performed, we carefully added 20 μL of fluorescent streptavidin at 0.4 mg mL^−1^ (S32354, Life Technologies) or non-fluorescent streptavidin at 2 mg mL^−1^ (85878, Sigma Aldrich) to the microspheres through the porous membrane. After 30 min, the sample chamber was washed with distilled water to remove the free streptavidin, and spectral acquisition was performed on the same microspheres.

For the preparation of liquid-crystal reflectophores, 4 μL of 5CB nematic liquid crystal (328510, Sigma Aldrich) was mixed with 0.2 g of polydimethylsiloxane (Sylgard 184, Dow Corning) and sandwiched between indium-tin-oxide glass (703192, Sigma Aldrich) with a coverslip as a spacer (140 μm in thickness) and incubated at room temperature (~23 °C) for 24 h for solidification. A 9-MHz sinusoidal signal from a function generator (DFG-8010, EZ digital) was amplified by a linear power amplifier (350L, Electronics & Innovation) to drive 0–1.5 V_rms_ μm^−1^ to the sample.

### Statistical analysis

We used GraphPad Prism for statistical analyses. Unpaired *t*-tests (two-sided) were used for group comparisons by assuming normality, based on previous literature. Neither randomization nor blinding was applied. For regression analysis, we present the correlation coefficient (*R*^2^) along with the sample size. Data are presented as either mean ± standard error or box-and-whisker plots, as otherwise indicated. We considered a *p*-value <0.05 to be statistically significant.

### Code availability

The MATLAB scripts used for data analysis (simulation database, spectrum fitting) are available at an open source repository (https://github.com/neurophotonic/reflectophore).

## Electronic supplementary material

Supplementary Information

Peer Review File

Reporting Summary

Source Data

## Data Availability

All relevant data are available from the corresponding author upon request. A reporting summary for this Article is available as a Supplementary Information file. The source data underlying Figs. 2f and 3c and Supplementary Figs. 7c and 11c are provided as a Source Data File.
