# Supplementary material for: Microsphere-based interferometric optical probe
Source: Nat Commun. 2018 Nov 1;9:4577. doi: 10.1038/s41467-018-07029-9 (PMC6212483; doi:10.1038/s41467-018-07029-9)
Supplement: Supplementary file 1 — Supplementary Information [file 41467_2018_7029_MOESM1_ESM.pdf]

Supplementary Information

Microsphere-based interferometric optical probe

Jo. et al.

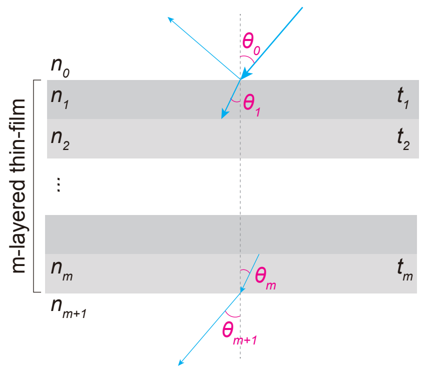

Supplementary Figure 1 | Generalized multilayered thin-film model. A multilayered thin-film composed of m-layers is presented. Arrows indicate incident, reflected, and refracted light waves. n_m, refractive index for the *m*-th thin-film layer. t_m, thickness for the m-th thin-film layer. θ_m, incident angle at the interface of m- and (m +1)-th layer. For most of our studies, the model is simplified to a mono-layered thin-film (m = 1) with a normal incident angle (θ = 0). For molecular sensing, the model has two additional layers (m = 3) formed by molecular adhesion.

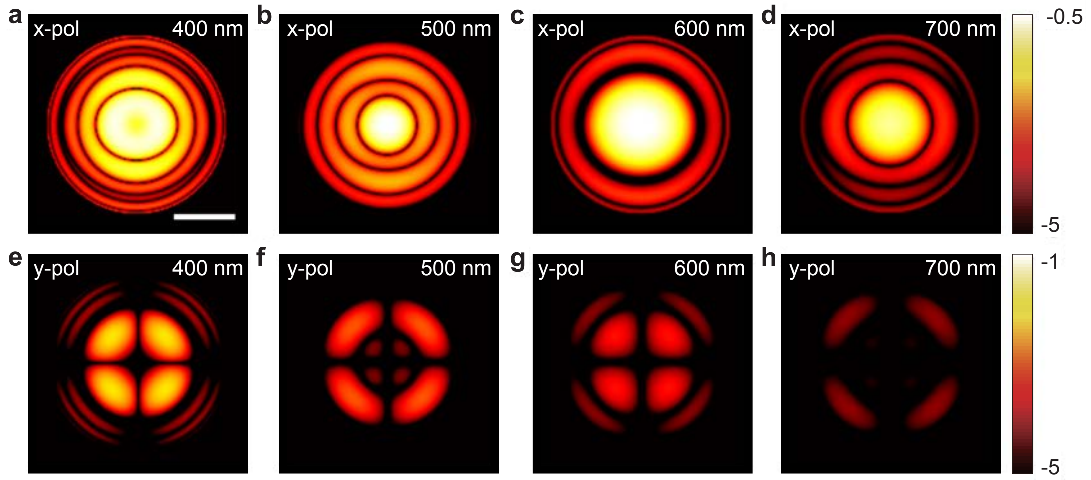

Supplementary Figure 2 | Vector diffraction model. Intensity distribution at the centroid of reflectophore is simulated with the vector diffraction model in an optical system with 1.1 NA. (**a-d**) Intensity maps for x-polarized field at indicated wavelengths. (**e-h**) Intensity maps for y-polarized field at indicated wavelengths. Colorbar indicates intensity in log scale. Scalebar, 300 nm.

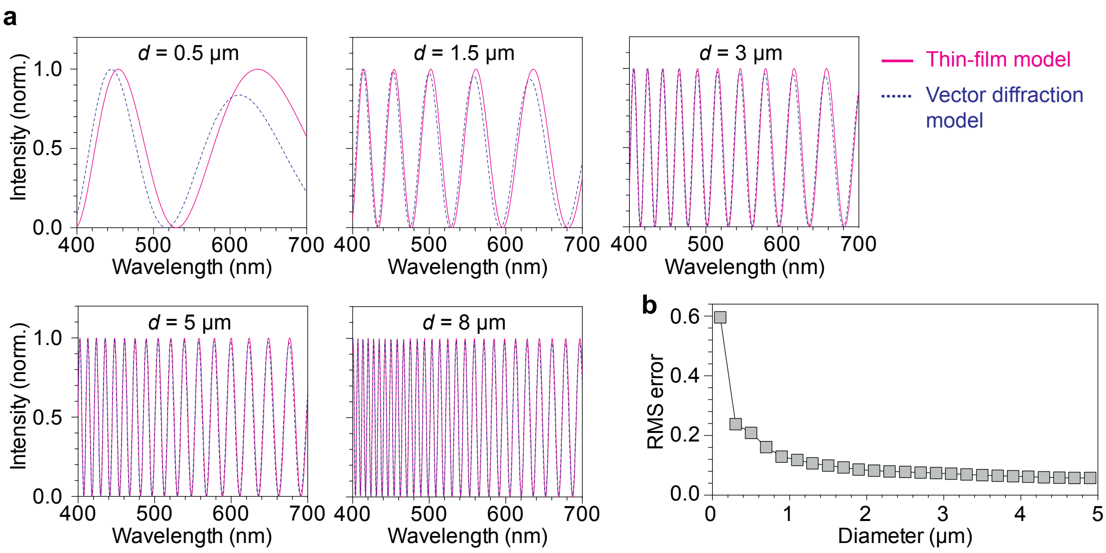

Supplementary Figure 3 | Validation of the thin-film model. (**a**) Representative simulation results. The thin-film model is compared with the vector diffraction model for various diameters of reflectophores. (**b**) Root-mean-squared (RMS) error between the two models.

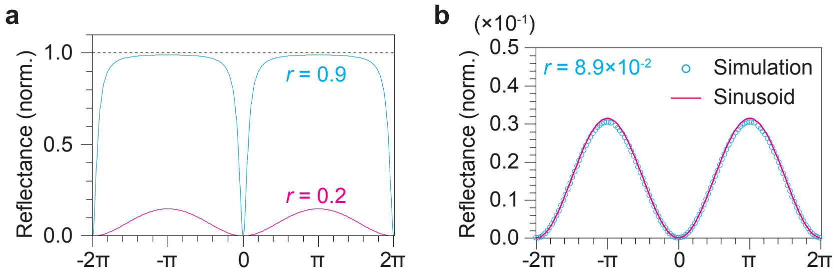

Supplementary Figure 4 | Sinusoidal approximation of reflectance spectra. (**a**) Reflectance intensity of mono-layered thin-film. Reflectance spectrum approximates to sinusoid if amplitude reflection coefficient is small ($r \ll 1$). (**b**) The reflectance spectrum of a polystyrene microsphere ($r = 8.9\times10^{-2}$) with a sinusoidal fitting.

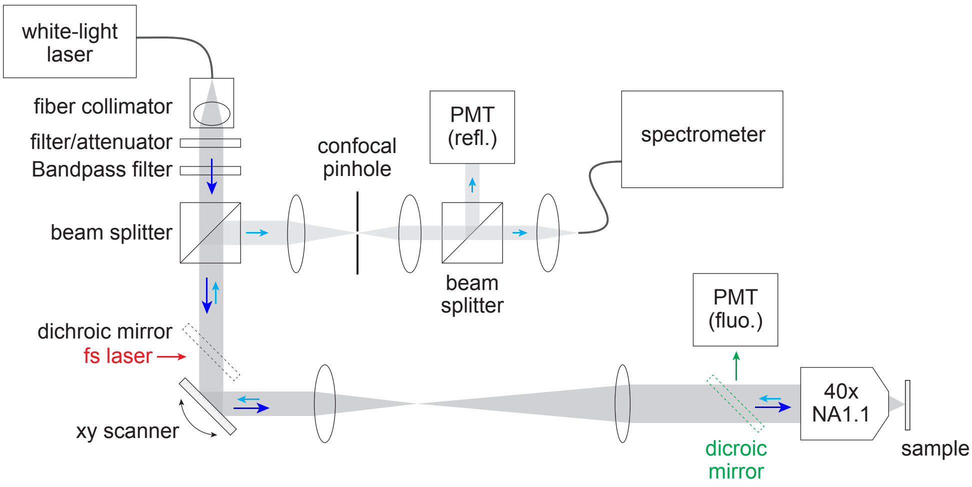

Supplementary Figure 5 | Optical setup. Reflectance-mode white-light interferometry setup coupled to two-photon fluorescence microscopy is constructed by modifying an upright microscopy. The system has 2 light sources, white-light laser for reflectance and femtosecond (fs) laser for two-photon fluorescence. There are 3 detection channels: a non-descanned PMT (photomultiplier tube) for two-photon fluorescence (fluor.), a descanned PMT for reflectance (refl.) imaging, and a spectrometer for reflectance spectrum (SpeRe). For switching between SpeRe and two-photon fluorescence, filter cube located near the objective back-aperture is rotated. NA, numerical aperture.

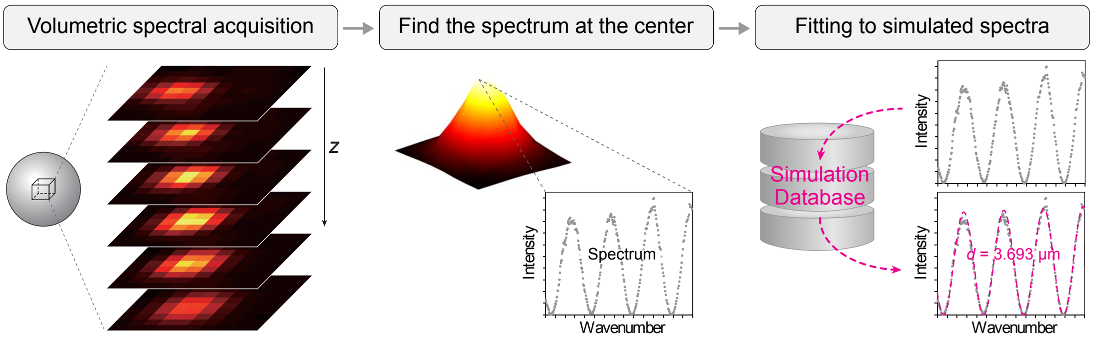

Supplementary Figure 6 | Step-by-step protocol for data acquisition and analysis. First, we acquire spectral information with volumetric scanning. Scanning dimension is typically 9×9×10 in xyz (Δ_{xy} = 100 nm, Δ_z = 200 nm). Second, we extract the spectrum having the maximal intensity, which corresponds to the geometric center of the microsphere. Third, we search for the simulated spectrum that shows the best-fit to the acquired spectrum to obtain the diameter of the reflectophore.

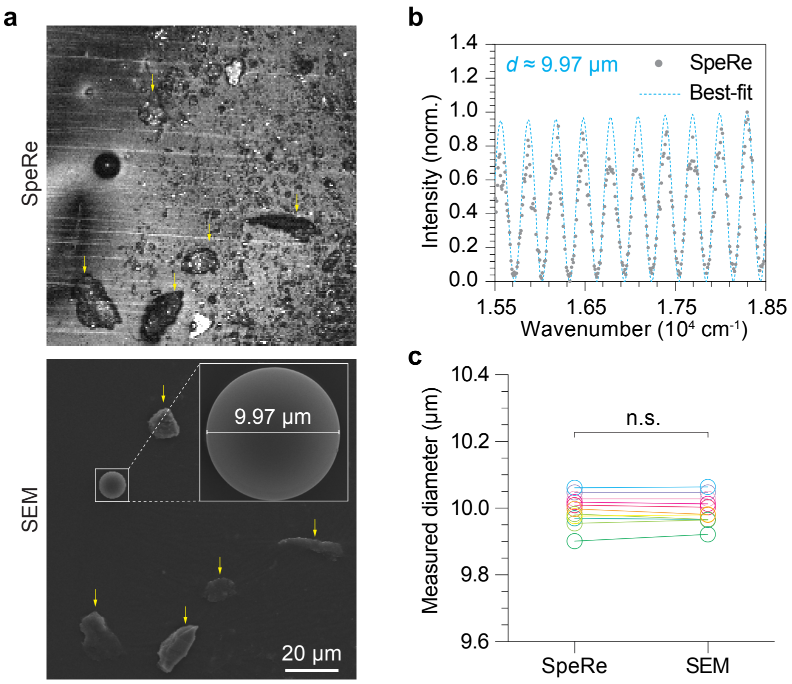

Supplementary Figure 7 | Correlative SEM-SpeRe imaging. (**a**) A representative correlative spectral reflectance (SpeRe) and scanning electron microscope (SEM) imaging. Arrows indicate dusts utilized as landmarks. (**b**) SpeRe analysis of the reflectophore in (a) (R^2 = 0.96). (**c**) Summary statistics (paired t-test, p = 0.735, n = 11 beads). Averaged absolute error was ~7.7 nm. n.s., not significant. Source data are provided as Supplementary Data 3.

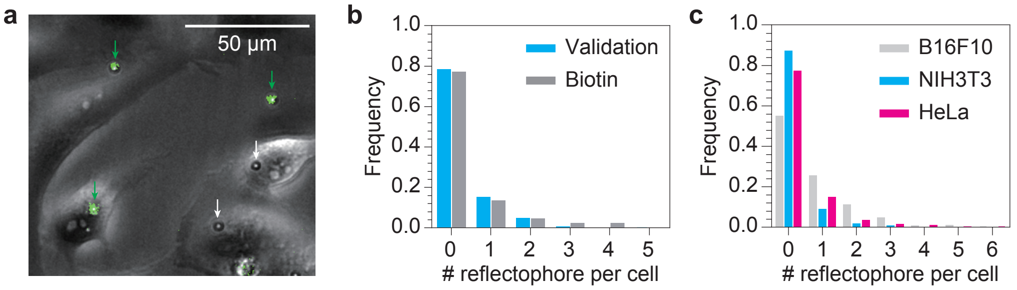

Supplementary Figure 8 | Efficiency of endocytosis by surface coating. (**a**) Validation of endocytosed biotin-coated reflectophores. Fluorescent streptavidin (green) is added to culture media, which only stains biotin-coated reflectophores at extracellular space. White and green arrows indicate the endocytosed and extracellular reflectophores, respectively. (**b**) Quantification of the endocytosed reflectophores. The result obtained by fluorescent streptavidin (0.30±0.028 reflectophores per cell, n = 556 cells) agrees well with the measurement by the phase-contrast microscope (0.39±0.13 reflectophores per cell, n = 44 cells; unpaired t-test, p = 0.51). (**c**) Quantification of intracellular reflectophores on three cell lines (B16F10: 0.786 reflectophores per cell, n = 1089 cells; NIH3T3: 0.197 reflectophores per cell, n = 918 cells; HeLa: 0.381 reflectophores per cell, n =754 cells).

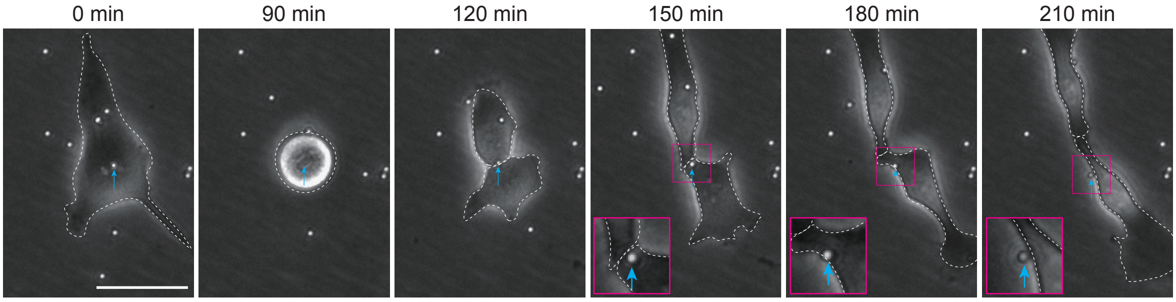

Supplementary Figure 9 | Time-series phase-contrast images on exocytosis of a microsphere. Dashed line demarcates the cell margin. The arrows indicate the same microsphere of interest. Inset, 2X magnified view of the indicated region. Scalebar, 40 μm.

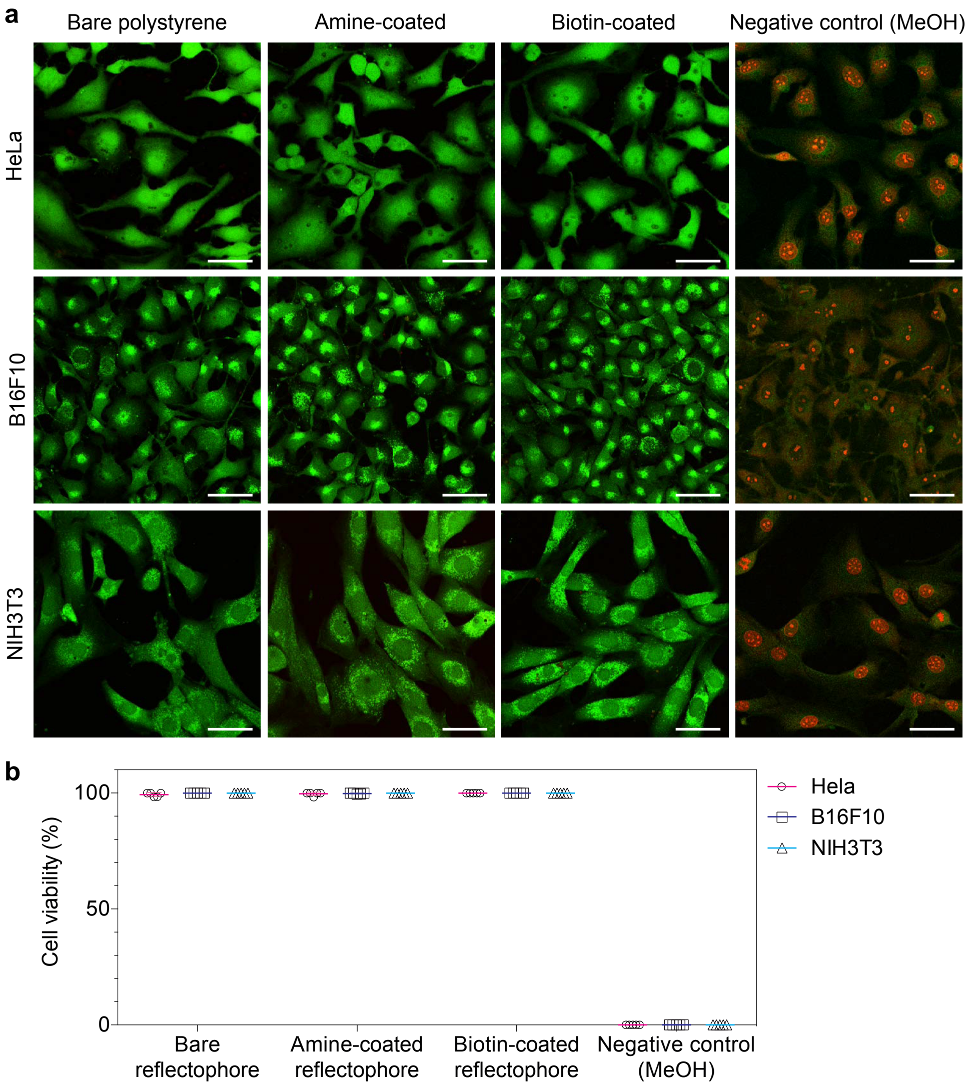

Supplementary Figure 10 | Cell viability test. (**a**) Representative images after 24 hours of incubation. Green (calcein AM) and red (ethidium homodimer-1) fluorescence represents viable and dead cells, respectively. Scalebar, 50 μm. (**b**) Quantification.

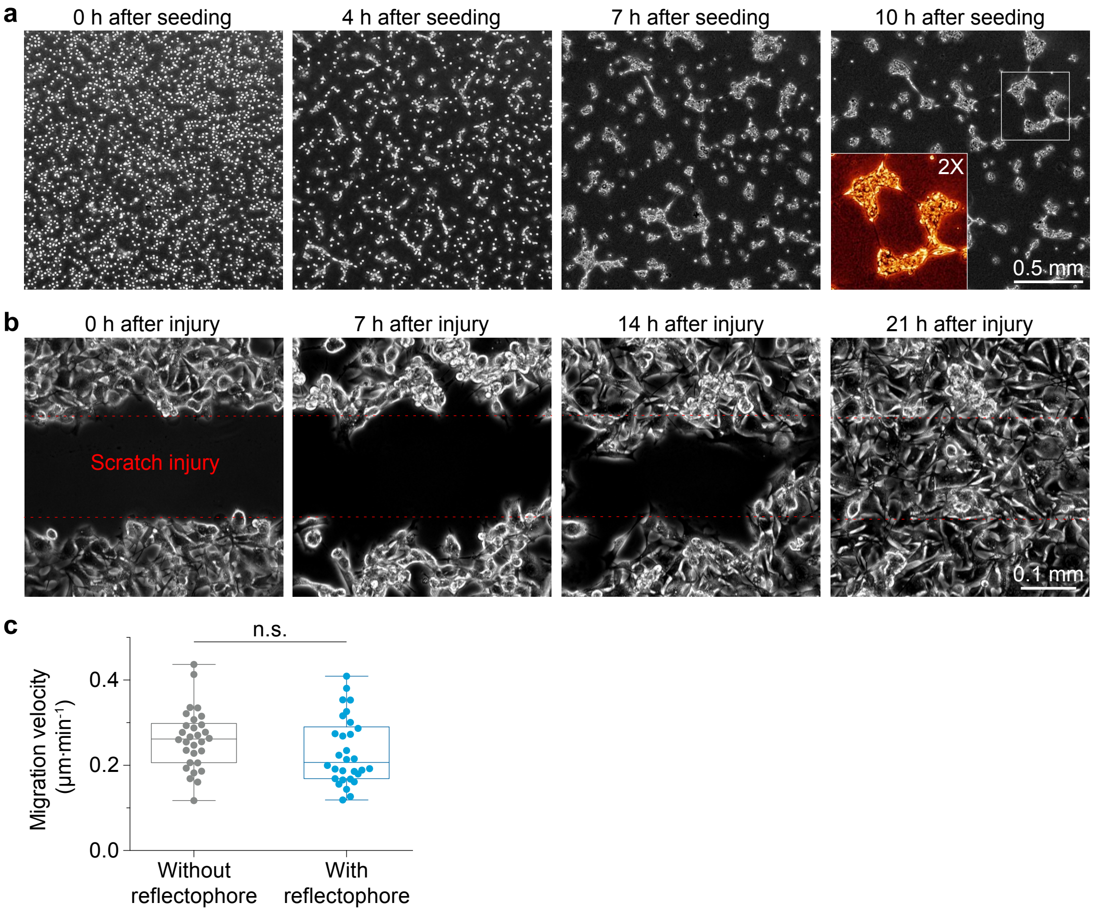

Supplementary Figure 11 | Migration assay. (**a**) Representative time-series images of capillary-formation in B16F10 cells cultured on Matrigel. (**b**) Scratch-injury assay to compare migration activity in cells with and without reflectophores. (**c**) Migration velocity of cells with and without reflectophores. There is no significant difference between two groups (0.261±0.013 μm·min^{-1} for 'without reflectophore', n = 30 cells; 0.232±0.014 μm·min^{-1} for 'with reflectophore', n = 30 cells; unpaired t-test, *p* = 0.139). Box-and-whisker plots show median, 25th and 75th percentiles, and minimum and maximum values. n.s., not significant. Source data are provided as Supplementary Data 4.

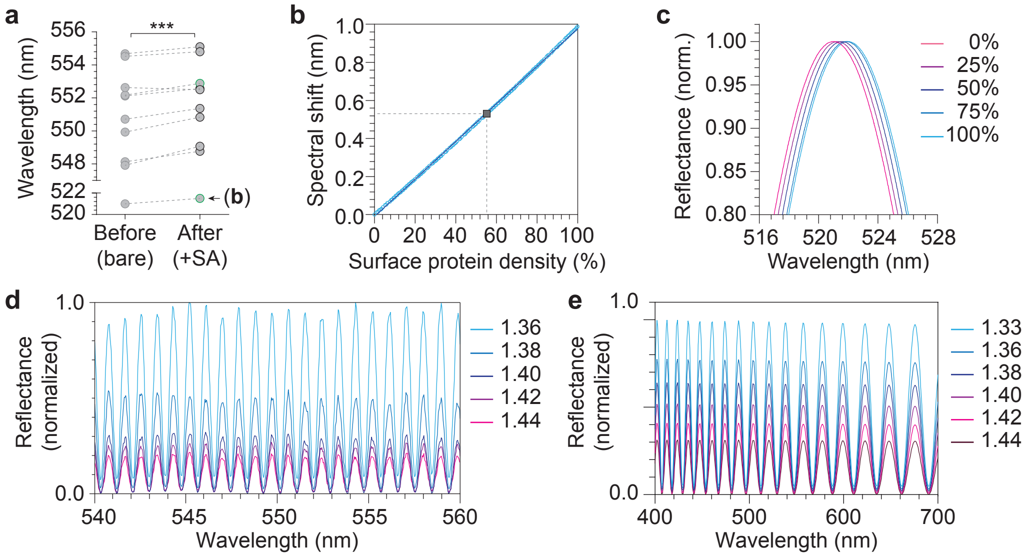

Supplementary Figure 12 | Spectral change by protein adhesion and external media. (**a**) Spectral shift by the binding of streptavidin (SA) to biotin-coated reflectophores (paired t-test; ***, $p = 0.0003$, n = 11 beads). Overall peak shift was 0.57±0.086 nm. Green and black outlines indicate fluorescent and non-fluorescent streptavidin, respectively. (**b**) Degree of spectral shift with respect to the surface density of streptavidin. The square point indicates the spectral shift in (a) and the corresponding surface protein density of 55% ($R^2 = 0.9997$). (**c**) Simulated reflectance spectrums with respect to the surface protein density. (**d**) Reflectance spectrum of a 100 μm polystyrene reflectophore with respect to the external media. Refractive index of the externa media is changed by increasing sucrose concentration in water from 1.36 (0.63 M) to 1.44 (2.3 M). Note that there is change in amplitude but not in peak position. (**e**) Numerical vector diffraction simulation on a 5 μm polystyrene reflectophore. Refractive index of the externa media is changed from 1.33 to 1.44.

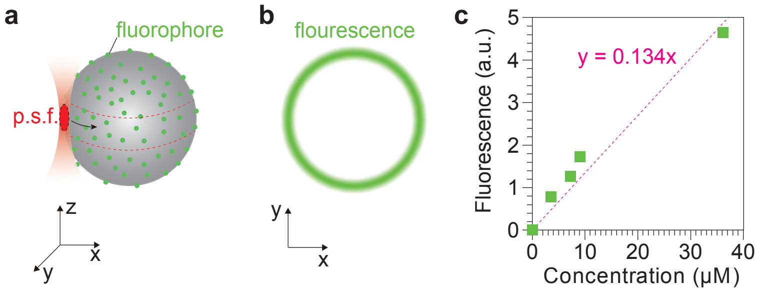

Supplementary Figure 13 | Fluorescence-based estimation of the surface protein density. (**a**) A schematic drawing of two-photon excitation on a reflectophore coated with a fluorophore. Red dashed lines demarcate the excited volume by horizontally scanning the point-spread-function (p.s.f.). (**b**) Horizontal cross-sectional view of the fluorescence emission. (**c**) A calibration curve for 'Alexa 488'-conjugated streptavidin (R^2 = 0.95).

Supplementary Note 1. Simulation

Numerical simulation for thin-film interference is performed based on transfer matrix theory[1,2]. The simulation parameters include layer thickness (d_i), incidence angle of light (θ_i), and refractive index of each layer (n_i) (Supplementary Fig. 1). Absorption coefficient is considered by incorporating the imaginary part of refractive index. The thin-film interference transfer matrix for multilayers is described as below.

$$M = \begin{pmatrix} M_{11} & M_{12} \\ M_{21} & M_{22} \end{pmatrix} = J_0^{-1}\left(\prod_{i-1}^{m} H_i\right) J_m \tag{1}$$

where, J_i and H_i defined as

$$J_i = \begin{pmatrix} s_i & s_i \\ t_i & -t_i \end{pmatrix}, \qquad where\ i = 1, \dots, m+1 \tag{2}$$

$$H_i = \begin{pmatrix} \cos(kn_i d_i \cos\theta_i) & i\sin(kn_i d_i \cos\theta_i)\dfrac{s_i}{t_i} \\ i\sin(kn_i d_i \cos\theta_i)\dfrac{t_i}{s_i} & \cos(kn_i d_i \cos\theta_i) \end{pmatrix}, \qquad where\ i = 1, \dots, m \tag{3}$$

with $s_i = 1, t_i = n_i \cos\theta_i$ for TE-wave or $s_i = \cos\theta_i, t_i = n_i$ for TM-wave. Hence, total reflectance (R) and transmittance (T) is described as follows.

$$R = \left|\frac{M_{21}}{M_{11}}\right|^2 \tag{4}$$

$$T = \frac{n_{m+1}}{n_0}\frac{\cos\theta_{m+1}}{\cos\theta_0}\left|\frac{1}{M_{11}}\right|^2 \tag{5}$$

To further validate the thin-film model, simulation based on vector diffraction theory was performed[3]. The vector-form complex field of focused wave in a high NA system is given by

$$\mathbf{E}_{\mathrm{foc}}(\mathbf{r}) = -\sum_{u=x,y,z}\sum_{w=S,P}\sum_{v=x,y}\frac{if}{\lambda}\iint_0^{NA} P_{vwu}(\alpha,\beta)\, A_v(\alpha,\beta)\frac{\mathrm{e}^{-i\mathbf{k}\cdot\mathbf{r}}}{\gamma}\,\mathrm{d}\alpha\mathrm{d}\beta\,\hat{\mathbf{u}} \tag{6}$$

where $P_{vwu}(\alpha,\beta)$ is the component of polarization decomposition matrix to describe local polarization variation[4], $A(\alpha,\beta)$ is electric field at the pupil plane of the objective lens, α, β, γ are direction cosines which are related to the wavevector, $\mathbf{k}$.

$$\mathbf{k} = (k_x, k_y, k_z) = \frac{2\pi n}{\lambda}(\sin\theta\cos\varphi, \sin\theta\sin\varphi, \cos\theta) = \frac{2\pi n}{\lambda}(\alpha,\beta,\gamma) \tag{7}$$

where, $\alpha^2 + \beta^2 + \gamma^2 = 1$. The focused wave interacts with spherical surface of reflectophore and give rise to interference constructively or destructively with respect to the optical path inside the cavity. The complex field of light interacted with a spherical object, $O_w(\alpha,\beta,n,r_o)$, is expressed as

$$\mathbf{E}_{\mathrm{int}}(\mathbf{r}) = -\sum_{u=x,y,z}\sum_{w=S,P}\sum_{v=x,y}\frac{if}{\lambda}\iint_0^{\mathrm{NA}} O_w(\alpha,\beta,n,r_o)\, P_{vwu}(\alpha,\beta)\, A_v(\alpha,\beta)\frac{\mathrm{e}^{-i\mathbf{k}\cdot\mathbf{r}}}{\gamma}\mathrm{d}\alpha\mathrm{d}\beta\,\hat{\mathbf{u}} \tag{8}$$

where *n* is refractive index, r_0 is radius of reflectophore. The intensity distribution of light

interaction at the focal plane for a polystyrene reflectophore (r_0 = 500 nm) was visualized in Supplementary Fig. 2. Encircled energy in the focal volume is over 85%, which is related to detection sensitivity of the optic system.

Supplementary Note 2. Sinusoidal approximation

The total reflectance at a monolayer is described as follows.

$$\frac{\text{Reflected intensity}}{\text{Incident intensity}} = \frac{I_r}{I_i} = \frac{F\sin^2(\delta/2)}{1 + F\sin^2(\delta/2)} \tag{9}$$

δ is described as $\delta = (4\pi d/\lambda)\sqrt{n^2 - n_e^2 \sin^2\theta}$, where$\theta$, n and n_e is an incident angle, refractive indices of the thin-film and the external medium, respectively[5]. F is finesse, described as $F = (2r/1 - r^2)^2$, where r is amplitude reflection coefficient at normal incidence. When finesse is small ($F \ll 1$ i.e. $r \ll 1$), the total reflectance (Eq. 9) can be approximated to a sinusoidal function as follows.

$$\frac{I_r}{I_i} = \frac{F\sin^2(\delta/2)}{1 + F\sin^2(\delta/2)} \approx F\sin^2(\delta/2) \tag{10}$$

In the case of a polystyrene reflectophore immersed in water, finesse (F) is 3.22×10^{-2}, which is small enough to apply the sinusoidal approximation (Eq. 10). Indeed, a simulated spectrum showed near-complete overlap with sinusoidal function (Supplementary Fig. 4).

Supplementary Note 3. Brightness

To compare relative brightness between fluorescence and reflectance, we assumed a fluorescent polystyrene-based microsphere having a diameter of 3 μm and containing 100 μM fluorescein. Fluorescence emission from the focal point is described by the absorbed photons in the focal volume multiplied by the quantum yield (Φ). For the simplicity, we assumed a cylindrical point-spread-function with a cross-sectional area, A_{xy}. Fluorescence efficiency is defined as follows:

$$\text{Fuorescence efficiency} := \frac{\text{Emission intensity}}{\text{Excitation intensity}} = \frac{\phi\sigma N}{A_{xy}} \tag{11}$$

where N is the number of fluorophores in the focal volume. In our optic setup (NA = 1.15), diffraction-limited resolution is ~200 nm in lateral dimension and ~450 nm in axial dimension[6]. Fluorescein has absorption cross-section (σ) of 0.0121 nm^2 and quantum yield (Φ) of 0.92[7,8]. The resulting fluorescent efficiency is ~0.02%. Accounting for the collection efficiency of our optic system (NA = 1.15), we expect to obtain fluorescence light corresponding to ~0.005% of the excitation light. By contrast, a single reflectophore typically generates reflectance of ~3.5%, which is >500-fold higher efficiency than fluorescence.

Supplementary Note 4. Photothermal damage

We calculated photothermal damage by excitation on a 3 μm reflectophore immersed in water. The heat is produced by the absorption of input laser and is removed mainly by convective heat transfer to the external water medium. For the simplicity, we assumed that the temperature distribution in the reflectophore is spatially homogeneous. By the lumped capacitance model, temperature of the reflectophore, $T(t)$, is described as follows[9].

$$mC_p\frac{\mathrm{d}T(t)}{\mathrm{d}t} = \alpha dI - \pi d^2 h(T(t) - T_\mathrm{w}) \tag{12}$$

where m is mass, C_p is heat capacitance measured at constant pressure, I is laser intensity, α is attenuation coefficient, d is diameter, h is heat transfer coefficient, and T_w is temperature of water. Solving differential equation yields,

$$T(t) = T_\mathrm{w} + \frac{\alpha dI}{\pi d^2 h}(1 - \mathrm{e}^{-\frac{\pi d^2 h}{mC_p}t}) \tag{13}$$

The parameters are as follows: m = 1.47×10^{-11} g; C_p = 1.22 J·g^{-1}·K^{-1}, α = 6.28×10^{-6} nm^{-1}, h is 3.94×10^{5} W·m^{-2}·K^{-1} [10–12]. The steady-state temperature in the reflectophore is reached in ~10 μs and the temperature increase is ~0.03 °C at a typical input power of ~20 μW. It is orders-of-magnitude lower than the glass transition temperature of the polystyrene (98 °C)[13].

Supplementary Note 5. Estimation of surface protein density

To estimate the surface density of streptavidin on a biotin-coated microsphere, we first obtained the calibration curve for fluorescence intensity with respect to the concentration of streptavidin (Supplementary Fig. 13). Using the diffraction-limited focal volume (4.63×10^{7} nm^{3}) [14], we estimated that the brightness of a single streptavidin molecule is 4.8×10^{-3} a.u. Using this information, a single-section fluorescence image (in a.u.) is converted to the number of molecules. By applying the hydrodynamic size of the streptavidin molecule (~5 nm), we estimated that the cover density is ~45%, which is similar to our independent estimation by spectral peak-shift (~52%).

Supplementary References

1. Stavenga, D. G. Thin film and multilayer optics cause structural colors of many insects and birds. *Mater. Today Proc.* **1,** 109–121 (2014).
2. Katsidis, C. C. & Siapkas, D. I. General transfer-matrix method for optical multilayer systems with coherent, partially coherent, and incoherent interference. *Appl. Opt.* **41,** 3978–3987 (2002).
3. Richards, B. & Wolf, E. Electromagnetic Diffraction in Optical Systems. II. Structure of the Image Field in an Aplanatic System. *Proc. R. Soc. A Math. Phys. Eng. Sci.* **253,** 358–379 (1959).
4. Kwon, J. *et al.* Label-free nanoscale optical metrology on myelinated axons in vivo. *Nat. Commun.* **8,** 1832 (2017).
5. Hecht, E. *Optics*. (Addison Wesley, 2002).
6. Cole, R. W., Jinadasa, T. & Brown, C. M. Measuring and interpreting point spread functions to determine confocal microscope resolution and ensure quality control. *Nat. Protoc.* **6,** 1929–1941 (2011).
7. Josephson, L., Tung, C. H., Moore, A. & Weissleder, R. High-efficiency intracellular magnetic labeling with novel superparamagnetic-tat peptide conjugates. *Bioconjug. Chem.* **10,** 186–191 (1999).
8. Brouwer, A. M. Standards for photoluminescence quantum yield measurements in solution (IUPAC Technical Report). *Pure Appl. Chem.* **83,** 2213–2228 (2011).
9. Cengel, Y. A. *Heat transfer: A practical approach*. *Mc Graw-Hill* (2003).
10. Ma, H. & Tian, Z. Effects of polymer chain confinement on thermal conductivity of ultrathin amorphous polystyrene films. *Appl. Phys. Lett.* **107,** 073111 (2015).
11. Marti, E., Kaisersberger, E. & Moukhina, E. Heat capacity functions of polystyrene in glassy and in liquid amorphous state and glass transition: DSC and TMDSC study. *J. Therm. Anal. Calorim.* **85,** 505–525 (2006).
12. Whitaker, S. Forced convection heat transfer correlations for flow in pipes, past flat plates, single shperes, and for flow in packed beads and tube bundles. *AIChE J.* **18,** 361–371 (1972).
13. Rieger, J. The glass transition temperature of polystyrene. *J. Therm. Anal.* **46,** 965–972 (1996).
14. Zipfel, W. R., Williams, R. M. & Webb, W. W. Nonlinear magic: multiphoton microscopy in the biosciences. *Nat. Biotechnol.* **21,** 1369–1377 (2003).
