# Supplementary material for: Microsphere-based interferometric optical probe
Source: Nat Commun. 2018 Nov 1;9:4577. doi: 10.1038/s41467-018-07029-9 (PMC6212483; doi:10.1038/s41467-018-07029-9)
Supplement: Supplementary file 3 — Reporting Summary [file 41467_2018_7029_MOESM3_ESM.pdf]

nature research

Corresponding author(s): Myunghwan Choi

Reporting Summary

Nature Research wishes to improve the reproducibility of the work that we publish. This form provides structure for consistency and transparency in reporting. For further information on Nature Research policies, see Authors & Referees and the Editorial Policy Checklist.

Statistical parameters

When statistical analyses are reported, confirm that the following items are present in the relevant location (e.g. figure legend, table legend, main text, or Methods section).

n/a	Confirmed	
☐	☒	The exact sample size (*n*) for each experimental group/condition, given as a discrete number and unit of measurement
☐	☒	An indication of whether measurements were taken from distinct samples or whether the same sample was measured repeatedly
☐	☒	The statistical test(s) used AND whether they are one- or two-sided<br>*Only common tests should be described solely by name; describe more complex techniques in the Methods section.*
☒	☐	A description of all covariates tested
☒	☐	A description of any assumptions or corrections, such as tests of normality and adjustment for multiple comparisons
☐	☒	A full description of the statistics including central tendency (e.g. means) or other basic estimates (e.g. regression coefficient) AND variation (e.g. standard deviation) or associated estimates of uncertainty (e.g. confidence intervals)
☐	☒	For null hypothesis testing, the test statistic (e.g. *F*, *t*, *r*) with confidence intervals, effect sizes, degrees of freedom and *P* value noted<br>*Give P values as exact values whenever suitable.*
☒	☐	For Bayesian analysis, information on the choice of priors and Markov chain Monte Carlo settings
☐	☒	For hierarchical and complex designs, identification of the appropriate level for tests and full reporting of outcomes
☐	☒	Estimates of effect sizes (e.g. Cohen's *d*, Pearson's *r*), indicating how they were calculated
☐	☒	Clearly defined error bars<br>*State explicitly what error bars represent (e.g. SD, SE, CI)*

Our web collection on statistics for biologists may be useful.

Software and code

Policy information about availability of computer code

Data collection	'Prairie sofrware(Bruker)' and 'Andor SOLIS v4.28(Andor technology)' were used for controlling the scanner and spectroscopy, respectively.
Data analysis	Described in 'Optic simulation' and 'Code availability' in the Methods section.

For manuscripts utilizing custom algorithms or software that are central to the research but not yet described in published literature, software must be made available to editors/reviewers upon request. We strongly encourage code deposition in a community repository (e.g. GitHub). See the Nature Research guidelines for submitting code & software for further information.

Data

Policy information about availability of data

All manuscripts must include a data availability statement. This statement should provide the following information, where applicable:

- Accession codes, unique identifiers, or web links for publicly available datasets
- A list of figures that have associated raw data
- A description of any restrictions on data availability

Described in 'Data availability' in the Methods section.

Field-specific reporting

Please select the best fit for your research. If you are not sure, read the appropriate sections before making your selection.

☒ Life sciences ☐ Behavioural & social sciences ☐ Ecological, evolutionary & environmental sciences

For a reference copy of the document with all sections, see nature.com/authors/policies/ReportingSummary-flat.pdf

Life sciences study design

All studies must disclose on these points even when the disclosure is negative.

Sample size	We chose the sample size based on literatures in the field to validate accuracy of our technique.
Data exclusions	If the geometric center was not included within the scanning volume during the SpeRe measurement, the dataset was excluded from the analysis ('Data analysis' in the Methods section).
Replication	Spectral measurements were repeated at least 10 times to verify the reproducibility. The reproducibility of the measurements was quantified as standard deviation or standard error of mean(n = 20 measurements of 1 bead for Fig. 2b,c; n = 15 measurements of 4 beads for Fig. 5e; n = 10 measurements of 11 beads for Fig. 6c and Supplementary Fig. 12a).
Randomization	N/A (no randomization was applied)
Blinding	N/A (all the data analyses were based on quantitative metrics)

Reporting for specific materials, systems and methods

Materials & experimental systems

n/a	Involved in the study
☒	☐ Unique biological materials
☒	☐ Antibodies
☐	☒ Eukaryotic cell lines
☒	☐ Palaeontology
☐	☒ Animals and other organisms
☒	☐ Human research participants

Methods

n/a	Involved in the study
☒	☐ ChIP-seq
☒	☐ Flow cytometry
☒	☐ MRI-based neuroimaging

Eukaryotic cell lines

Policy information about cell lines

Cell line source(s)	We purchased the HeLa, B16F10 and NIH3T3 cell-line from ATCC (American Type Culture Collection).
Authentication	The manufacturer (ATCC) ensured the authenticity by using morphology, karyotyping, and PCR based approach.
Mycoplasma contamination	The cell lines were not tested for mycoplasma contamination.
Commonly misidentified lines (See ICLAC register)	N/A (No commonly misidentified cell lines were used in this study.)

Animals and other organisms

Policy information about studies involving animals; ARRIVE guidelines recommended for reporting animal research

Laboratory animals	Male or female Thy1-YFP (C57BL/6; Jackson laboratory) transgenic mice aged 7-10 weeks old were involved in this study. (Described in 'Preparation of scattering samples' in the Methods section.)
Wild animals	N/A (No wild animals were involved in this study.)
Field-collected samples	N/A (No samples collected from field were involved in this study.)
