# Supplementary material for: Microsphere-based interferometric optical probe
Source: Nat Commun. 2018 Nov 1;9:4577. doi: 10.1038/s41467-018-07029-9 (PMC6212483; doi:10.1038/s41467-018-07029-9)
Supplement: Supplementary file 2 — Peer Review File [file 41467_2018_7029_MOESM2_ESM.pdf]

Editorial Note: This manuscript has been previously reviewed at another journal that is not operating a transparent peer review scheme. This document only contains reviewer comments and rebuttal letters for versions considered at *Nature Communications.*

REVIEWERS' COMMENTS:

Reviewer #1 (Remarks to the Author):

The authors have convincingly addressed my remaining technical concern -- and done so by adding data to the manuscript that I believe will be quite useful to many readers.

Overall, I find the work well suited for publication in Nature Communications and I am happy to recommend publication of the manuscript in its present form.
